# A novel surgical technique in transforaminal lumbar interbody fusion by the bone graft delivery device: evaluation of therapeutic effect in patients with minimally invasive spine surgery

**DOI:** 10.1186/s12893-022-01773-y

**Published:** 2022-10-26

**Authors:** Kai-shun Yang, Chih-Wei Chen, Ru-Bin Yau, Huang-Chien Liang, Ching-Chung Ko, Jinn-Rung Kuo, Chung-Ching Chio, Sher-Wei Lim

**Affiliations:** 1 Department of Spinal Surgery, First Affiliated Hospital of Dali University, Dali, Yunnan China; 2 Division of Neurosurgery, Department of Surgery, Chi Mei Medical Center, Tainan City, 710 Taiwan; 3 Department of Occupational Safety and Health/Institute of Industrial Safety and Disaster Prevention, College of Sustainable Environment, Chia Nan University of Pharmacy and Science, Tainan City, 717 Taiwan; 4 Department of Medical Imaging, Chi-Mei Medical Center, Tainan City, Taiwan; 5 Department of Materials Engineering, Ming Chi University of Technology, New Taipei City, Taiwan; 6 Department of Health and Nutrition, Chia Nan University of Pharmacy and Science, Tainan City, Taiwan; 7 Department of Medical Research, Chi-Mei Medical Center, Tainan City, Taiwan; 8 Department of Nursing, Min-Hwei College of Health Care Management, Tainan City, Taiwan

**Keywords:** MIS device, Minimally invasive surgery, Bone graft, Bone fusion

## Abstract

**Background:**

Transforaminal Lumbar Interbody Fusion (TLIF) is commonly associated with higher complications and longer operative time. This study aims to evaluate the effectiveness, safety, and usability of a novel minimally invasive surgery (MIS) bone graft delivery device.

**Methods:**

73 consecutive patients with lumbar spondylosis, degenerative disc disease, spondylolisthesis, scoliosis or trauma were enrolled in this randomized controlled trial. Group 1 comprised 39 patients treated with the novel MIS bone graft delivery device. Group 2 consisted of 34 patients treated with the conventional system. The primary objective of the study was the assessment of the amount of bone graft delivery using the device. The secondary objectives were the effect of the device on operative time, pain relief, disability improvement, and bone fusion grade.

**Results:**

Bone delivery amount was significantly higher in the MIS device group (6.7 ± 2.9 mL) compared to the conventional group (2.3 ± 0.5 mL), p < 0.001. Regarding the operation time, the MIS device group was associated significantly lower duration than the conventional group (p < 0.001). After a 3-month follow-up, 39.5% of the patients in the MIS device group and 3.5% of the patients in the conventional group were observed to achieve grade I fusion (complete fusion). There was a significant difference in fusion success rates (p < 0.01).

**Conclusion:**

The novel MIS bone graft delivery device was associated with successful bone delivery. Our MIS device provides promising modality with less operative time and higher bone fusion rates than conventional modalities.

*Trial Registration* This trial was retrospectively registered on ClinicalTrials.gov (Registration date: 11/19/2021; Registration number: NCT05190055).

**Supplementary Information:**

The online version contains supplementary material available at 10.1186/s12893-022-01773-y.

## Introduction

Lumbar interbody fusion (LIF) is increasingly recognized as a feasible and effective approach for the management of a wide range of spine disorders including degenerative lumbar diseases and spondylolisthesis [1]. It has been commonly indicated in patients with symptomatic low back pain and/or disability after failure of conservative management [2]. Historically, posterior lumbar interbody fusion (PLIF) was the gold standard approach for spine fusion; however, transforaminal lumbar interbody fusion (TLIF) has emerged as a popular variant of PLIF that is based on lateral access of the intervertebral body, complete discectomy, and bone graft placement transforaminal [3]. The TLIF poses several advantages over the traditional posterior approach including easier access, lack of ligament damage, and the need for a single unilateral incision to allow bilateral anterior column support [4, 5]. Previous reports demonstrated that TLIF is associated with lower risk of spinal nerve damage compared to the traditional posterior approach [6]. Additionally, with the introduction of minimally-invasive surgery (MIS), the intra- and postoperative outcomes of TLIF have improved significantly in terms of muscle injury, bleeding, hospital stay, and functional recovery [1].

The delivery of autologous bone graft or bone substitutes is a major concern during the TLIF approach. It is well-established that a sufficient amount of bone graft ≧ 50% of the cross-sectional area of the gap must be placed to achieve a successful interosseous fusion. Failure to do so has been shown to decrease the success of fusion and negatively affect clinical outcomes [7]. Yoo et al. has reported a significantly increased fusion rate in MIS-TLIF with the increased bone graft volume [8], however, the limited visual field and the minimal destruction in MIS often poses a challenge to the amount of bone graft delivered. The minimal amounts of bone graft transplanted not only compromises the fusion rate but also can lead to increased intraoperative time and a higher risk of iatrogenic injury due to the repeated filling procedure [9]. Improved bone graft delivery tools allowing better visualization of the cannula tip and complete filling of the prepared disk space and thus reducing the risk accompanied by the repeated filling process has been published [10]. Shau et al. also demonstrated that an articulated delivery arm system for interbody graft placement facilitated increased segmental lordosis [11]. However, the inserted graft volume could be discrepant from the desired volume and was subjected to individual surgeons as delivering bone graft by these devices relied mainly on manual filling. The challenges remain as participants reported no significant improvement in the radicular pain and the fusion rate, which is directly related to the treatment efficacy, is left undetermined.

In light of the need to effectively deliver sufficient bone graft, we introduced a novel MIS bone graft delivery device (MIS device) with an integrated threaded rod. When connected to the surgical drill, it drives the granule bone graft into the filler tube and moving forwards during spinning. This MIS device allowed surgeons to directly deliver the graft into the deep side of patients in a safe, stable, continuous, and effective approach.

## Materials and methods

### Design and patients

After attaining the ethical approval from the Institutional Review Board of Chi Mei Medical Center (reference number: 10508-J01), patients were randomly allocated either to spinal surgery using the MIS device or the conventional modality. In this randomized controlled trial, a total of 73 participants with lumbar spondylosis, degenerative disc disease, spondylolisthesis, scoliosis, or trauma undergoing elective Transforaminal lumbar interbody fusion (TLIF) were recruited between 3/24/2015 and 12/6/2016. The follow-up period was 24 months for each participant. The eligible patients for this study should be above 18 years old with confirmed indication to TLIF through a posterior approach. Patients with an active infection, symptomatic osteoporosis, immature bone, pregnancy, active malignancy, and previous radiotherapy at the planned surgical site were excluded. Informed consent was signed by each participant before recruitment. We strictly followed the protocols of patient confidentiality and human subjects in the clinical trial implementation. The patients were blinded to the allocated surgical technique before the operation and during the follow-up period of 2 years.

### Surgical technique

**Fig. 1:**
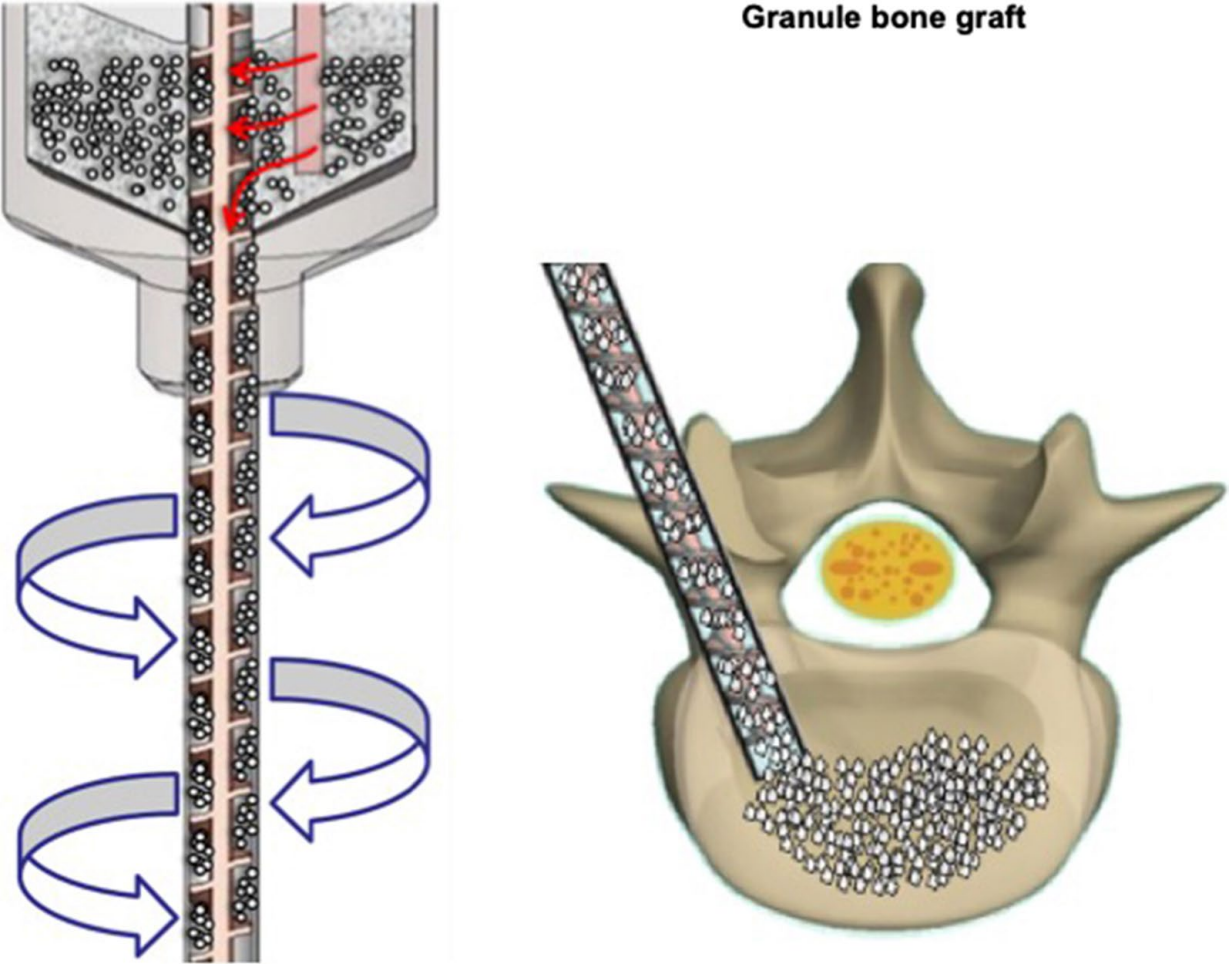
The bone graft delivery device. The novel device integrates the threaded rod into the filler tube allowing the granule bone graft to be driven deep into the empty disc space. The device can be connected with a surgical drill for rapid bone graft filling

Among the 73 patients, 34 were allocated to the control group and 39 to the MIS device group. After receiving prophylactic antibiotics according to the local hospital protocol, the patients were generally anesthetized in a prone position. Paramedian or midline posterior sections were conducted, exposing the lumber vertebras encompassing the facet joints. The facet joints were removed, and decompression was performed. The diseased disc nucleus was then excised before filling in the Bicera™ bone substitutes (Wiltrom Co. Ltd, Taiwan). For the control group, the bone substitutes were filled in the disc space manually using a bone grafting funnel. In the case of device group, a novel filler tube with integrated threaded rod, which can be connected to a surgical drill, was used for rapid and continuous graft filling (Fig. 1). The Bicera with its 0.5–1.0 mm particle size was then delivered at a speed of 3.2 ± 0.5 g per minutes with the surgical drill running at 600 rpm. A polyethyletherketone (PEEK) interbody fusion cage (Interbody fusion system-Space shuttle (Lumbar Cage)/PEEK 001 series or TLIF (Lumbar Cage)/PEEK 003 series for the conventional group and Interbody fusion system- Lumbar Cage/PEEK006 series for device group, Wiltrom Co. Ltd, Taiwan) was then placed into the disc space followed by fixation with pedicle screws (Spinal fixation system- 4030xx25-10 and 4031xx25-65, Wiltrom Co. Ltd, Taiwan) [12].

### Clinical and radiologic analysis

The treatment efficacy was assessed by clinical analysis based on the visual analogue scale (VAS) for radicular pain and the Oswestry Disability Index (ODI) questionnaire for low back pain. The VAS back and leg pain scores were measured preoperatively and postoperatively at 1, 3, 6, 12, and 24 months. The ODI questionnaires were taken preoperatively and postoperatively at 3, 6, 12, and 24 months. The bone graft delivery volume and the filling time during the surgery were measured to compare the efficiency of the two approaches.

The X-ray and computed tomography were performed prior to surgery and at 3-, 6-, 12-, and 24-months post-surgery for bone fusion assessment. Each image assessed by radiologist. The degree of bone fusion was classified into three grades. Grade I was defined as a complete fusion, union case, and grade II and III was defined as a partial union and a non-union case, respectively [13]. Bone fusion volume was further determined by three-dimensional reconstruction images using ITK-SNAP software (ITK-SNAP 3.8.0, http://www.itksnap.org).

### Statistical analysis

Data were analysed using SPSS version 23. Baseline characters were expressed by the interventional groups and compared using descriptive statistics. As descriptive tools, mean and standard deviation were presented for continuous variables. Continuous data were evaluated using *t*-test to compare mean changes between two groups. Statistical significance was set at p < 0.05.

## Results

### Baseline clinical data and outcome assessment

Among the 73 participants in the study, 34 patients received a conventional bone delivery approach, in which bone graft was manually filled in through a bone graft funnel, and 39 patients had the bone graft transplanted through the novel MIS bone delivery device (Table 1 and Additional file 1). The average age was 74.8 ± 6.4 and 74.5 ± 7.6 years in the control group and the MIS device group, respectively. No inter-group differences on male to female ratio and BMI levels were observed. The cage levels of bone grafting performed in the two groups were shown. Noticeably, patients in the MIS device group had significantly less blood loss (p < 0.05) during the surgery compared with the control group. Other perioperative parameters including operating time, time to post-op ambulation, and days of admission were comparable between the two groups.

**Table 1:** Baseline clinical data and perioperative parameters of patients who received TLIF using traditional bone delivery method (Control group) or the novel device (MIS device group)

	Control group		MIS device group		Statistic
Age (years)	74.8 ± 6.4		74.5 ± 7.6		p: 0.82
Sex (M: Male; F: Female)	M F	38.2% 61.8%	M F	38.5% 61.5%	p: 0.98
BMI (kg/m^2^)	25.3 ± 4.5		25.9 ± 3.1		p: 0.56
Operated level (s)					
1 level	17		11		
2 levels	12		20		
3 levels	5		7		
4 levels	0		1		
Level					
L2-3	5 (8.9%)		7 (9.2%)		
L3-4	14 (25.0%)		23 (30.3%)		
L4-5	27 (48.2%)		34 (44.7%)		
L5-S1	10 (17.9%)		12 (15.8%)		
Blood loss (mL)	99.4 ± 73.2		64.6 ± 50.1		p < 0.05
Operating time (min)	241.5 ± 117.6		229.7 ± 76.8		p: 0.31
Time to post-op ambulation (day)	1.8 ± 0.9		1.6 ± 0.6		p: 0.13
Days of admission (day)	6.4 ± 2.7		6.1 ± 2.8		p: 0.36

**Fig. 2:**
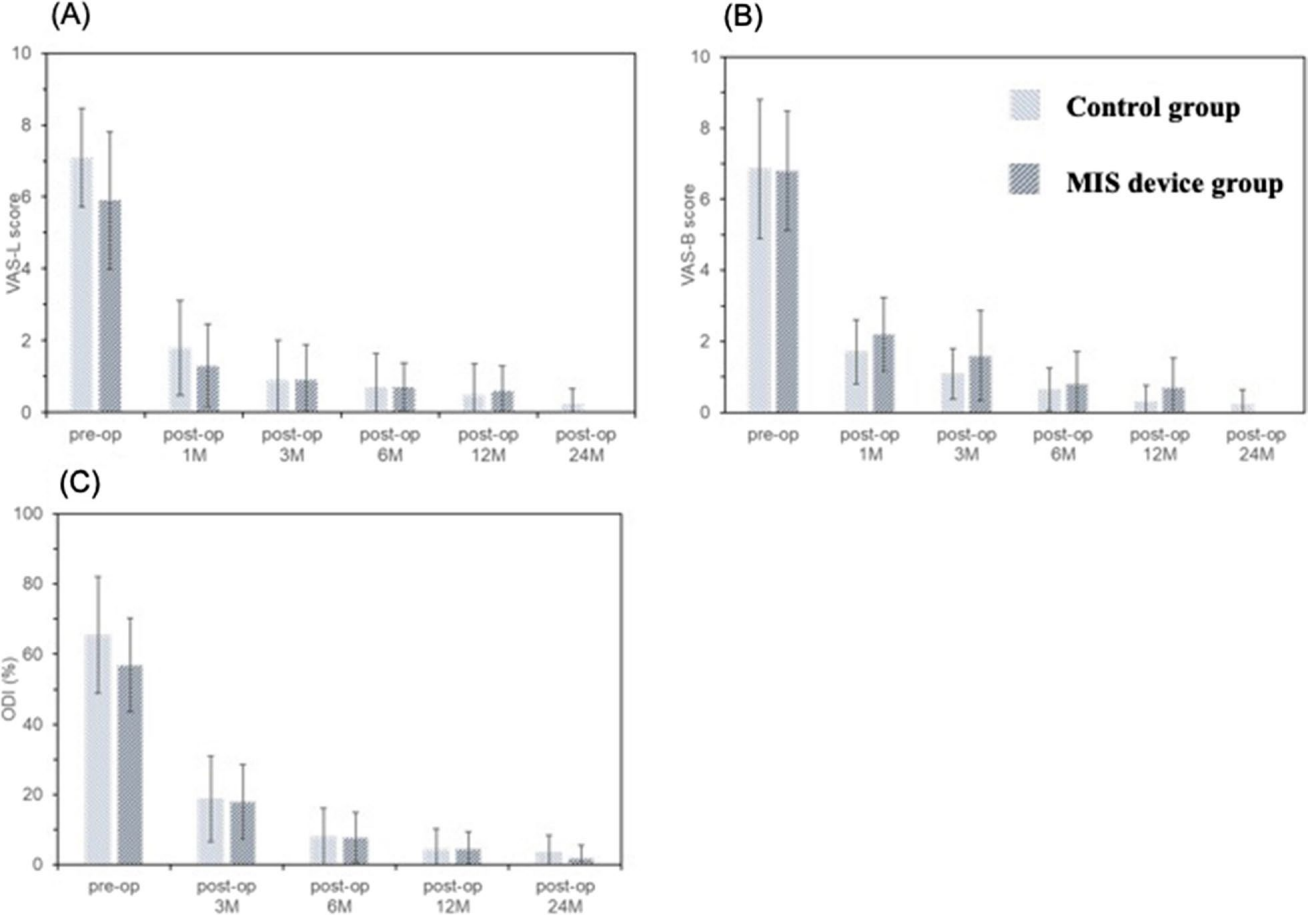
VAS and ODI in subjects receiving the 2 different graft filling techniques. Visual analogue scale (VAS) leg (**A**) and back (**B**) pain scores and Oswestry disability index (**C**) prior to and at each follow-up time points post operation in subjects receiving the 2 different graft filling techniques. There was no significant difference between the groups at each time point regarding VAS pain scores and ODI

The VAS and the ODI were employed to assess the surgery outcomes. Both the conventional bone graft delivery approach and the MIS device effectively reduced the radicular pain in patients’ back and leg after surgery (Fig. 2). VAS in back pain before the surgical operation was 6.9 ± 2.0 and 6.8 ± 1.8 in the control group and MIS device group, respectively. At 1 month post-surgery, VAS back pain score significantly decreased to 1.7 ± 0.9 and 1.8 ± 1.2 in the control group and the MIS device group. The score was further improved throughout the follow-up period. Additionally, VAS leg pain score showed a similar trend with minimal VAS in the MIS device groups at the final follow-up (24 months). For ODI assessment, TLIF treatment decreased ODI for more than 60% in both groups. Prior to the surgical operation, ODI was 65.4 ± 16.6% and 56.9 ± 13.4% in the control group and the MIS device group respectively, and then reduced to 18.8 ± 12.2% and 18.0 ± 10.6% at 3 months post-surgery. The ODI at final visit in the control group and MIS device group was 3.4 ± 5.1% and 1.9 ± 3.7%, respectively.

### Assessment of the bone delivery approaches

**Fig. 3:**
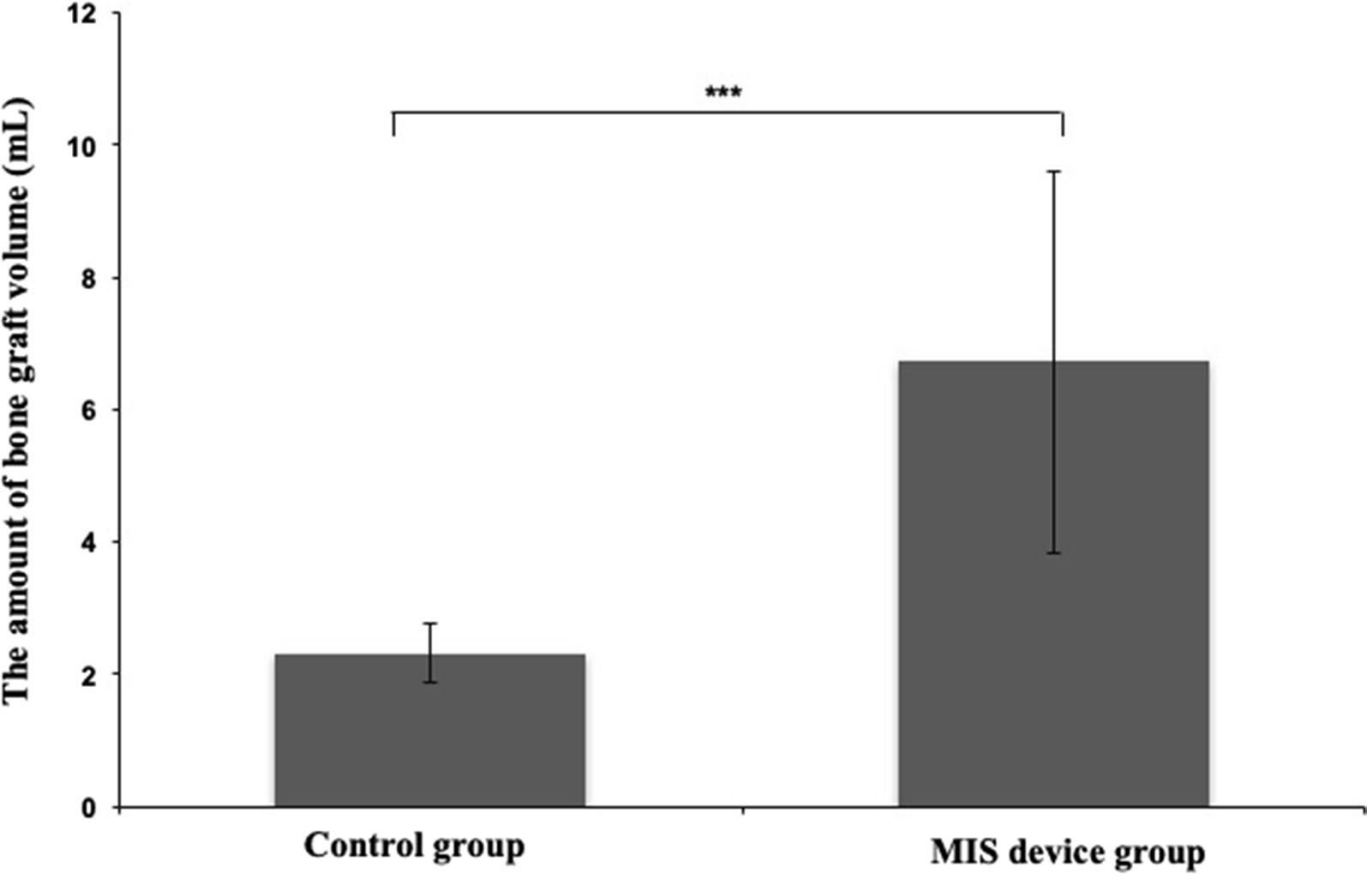
The amount of bone graft volume performed using Control group or MIS device group. The amount of bone graft volume performed using the traditional delivery method (Control group) or the novel device (MIS device group). The novel device significantly increased the volume of the bone graft material transplanted into the disc space. ***p < 0.001

The graft filling time was significantly reduced to 20 s in the MIS device group compared with that of 125 s in the conventional approach (Table 2). The use of this MIS device could deliver a significantly higher average bone graft volume of 6.7 ± 2.9 mL for transplantation and reach a maximum of 12.5 mL delivery volume in some cases (Fig. 3). While the conventional approach delivered 0–2.5 mL to 83.9% cases with an average 2.3 ± 0.5 mL delivery volume to all cases, the MIS device was able to transport > 2.5 mL to more than 90% cases. Interestingly, none of the cases received > 5.0 mL of graft volume using the conventional approach when more than 40% of cases received bone graft volume > 5.0 mL using the MIS device.

**Table 2:** Average bone graft filling time and graft volume transplanted in each case

	Control group		MIS device group	
Bone graft filling time (s)	125 ± 6.8		20 ± 0.2	
Bone graft volume (mL)				
0–2.5	47	(83.9%)	7	(9.2%)
2.5–5	9	(16.1%)	35	(46.1%)
5–7.5	0	(0.0%)	8	(10.5%)
7.5–10	0	(0.0%)	19	(25.0%)
10–12.5	0	(0.0%)	7	(9.2%)
	2.31 ± 0.45 ml		6.72 ± 2.88 ml

**Fig. 4:**
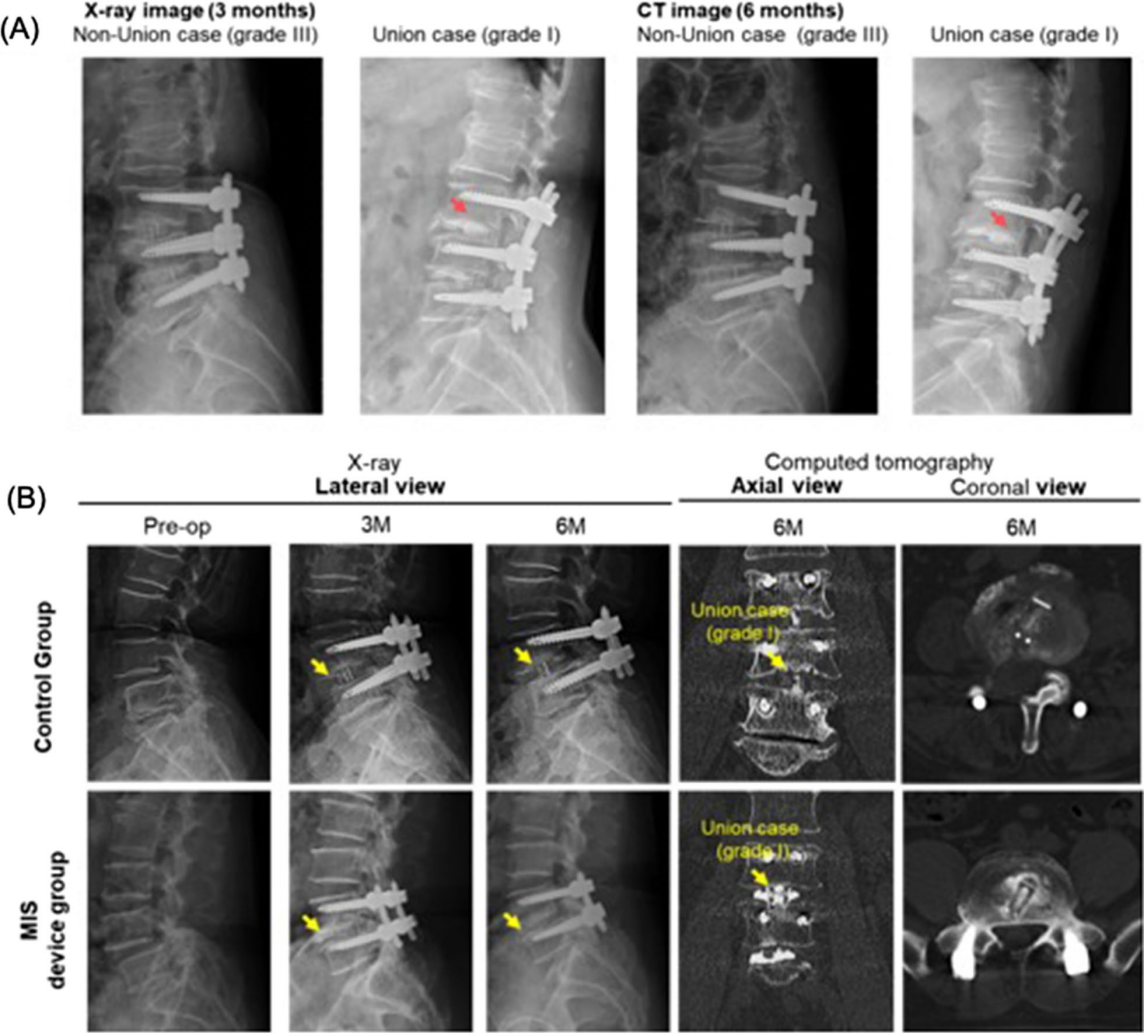
Representative radiography and computed tomography of surgical area of participant’s non-union and union cases. Representative radiography and computed tomography of surgical area of participant’s non-union and union cases. **A** X-ray image of the non-union and union case. Bone bridge (red arrow) was observed in the union case on X-ray taken 3 months post operation and the bone graft surrounding the cage (red arrow) was seen on X-ray taken 6 months after the surgery. **B** X-ray and CT images of the union cases receiving traditional or MIS graft delivery method. Bony formation and incorporation are indicated in yellow arrows

**Fig. 5:**
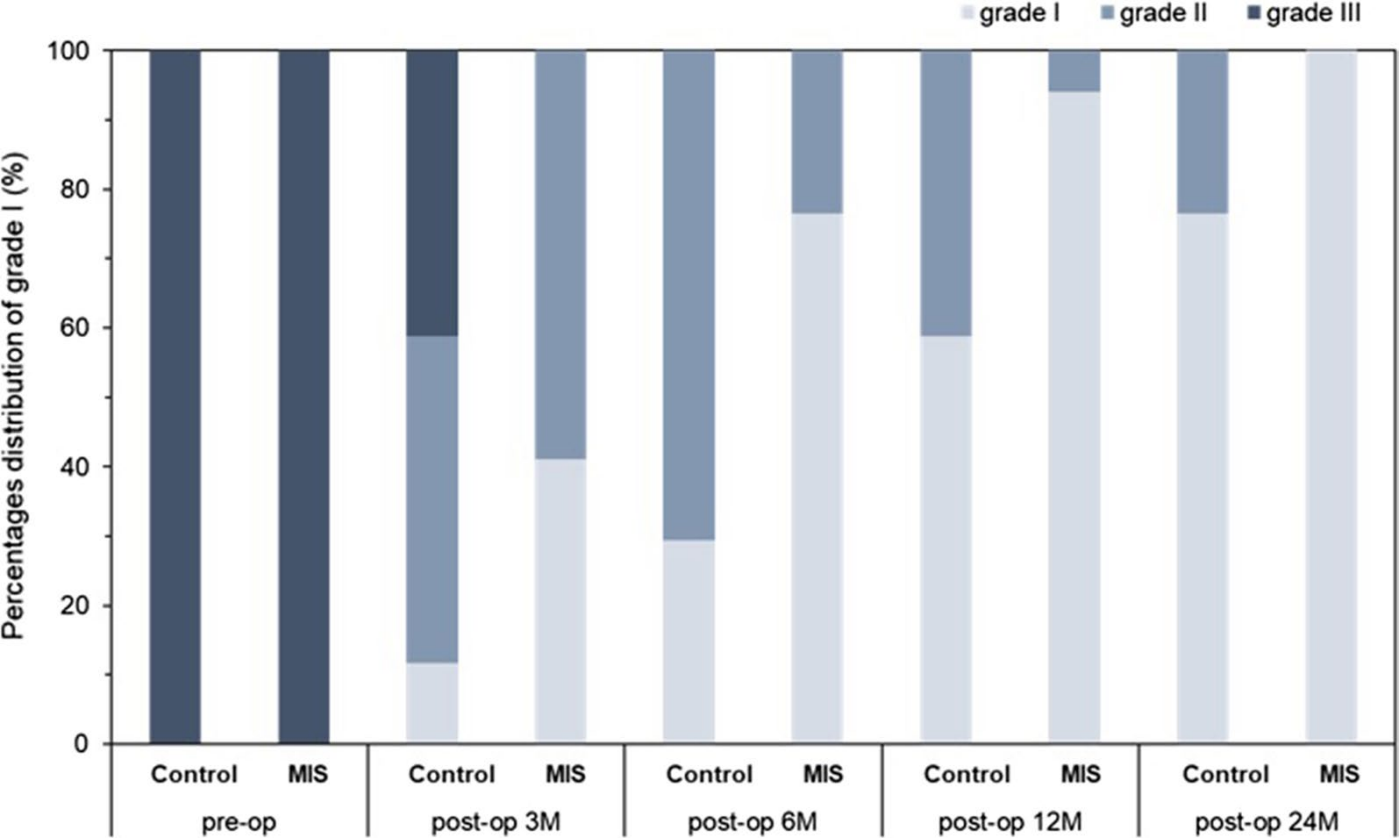
The percentage distribution of grade fusion at each follow-up time points. The percentage distribution of grade I, II, and III fusion prior to and at each follow-up time points in cases with the traditional method or novel device. Grade I, II, and III indicates complete fusion, partially fusion, and no fusion of the cases

**Fig. 6:**
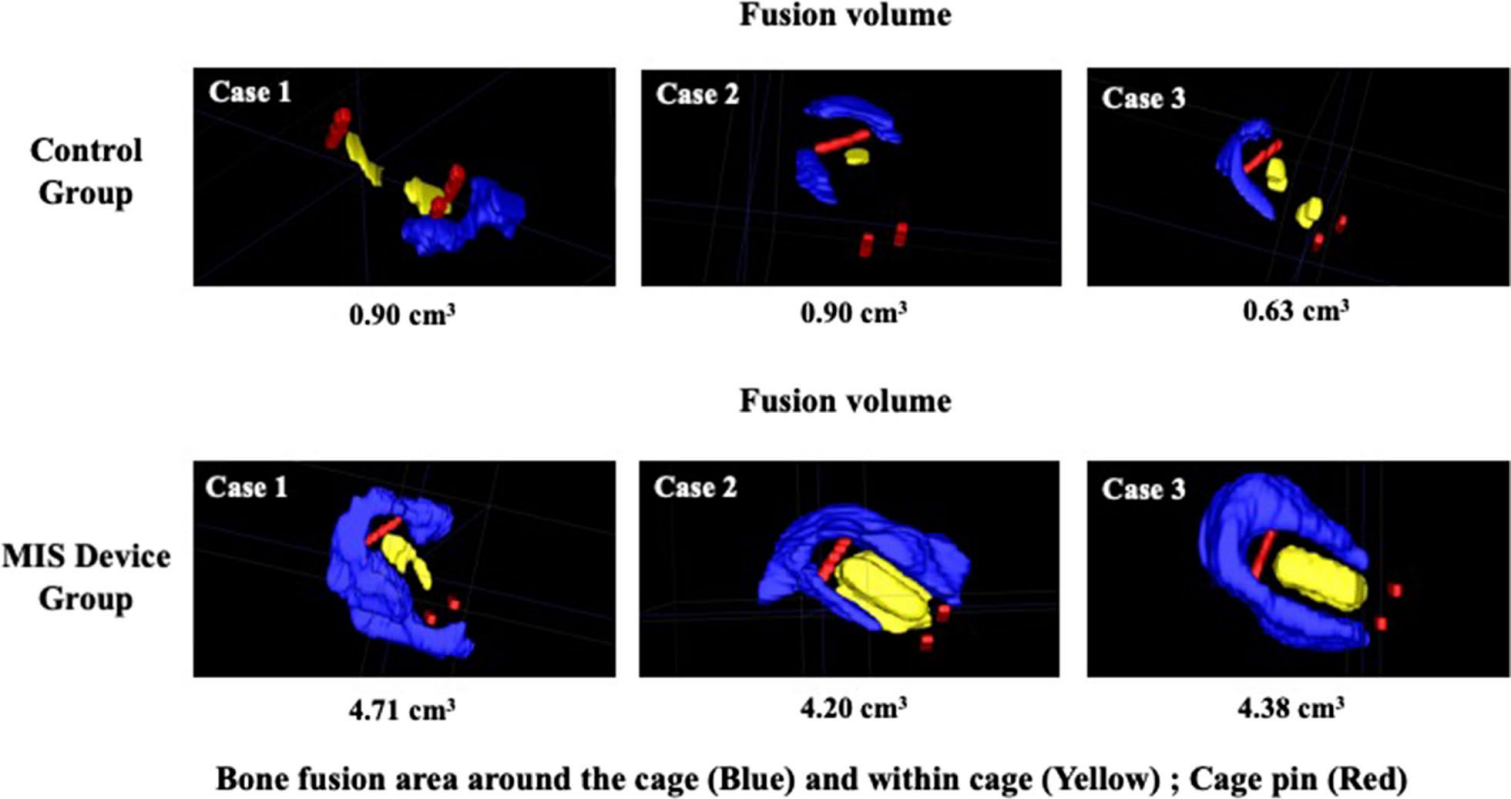
Representative three-dimensional reconstruction computed tomography of bone fusion volume at 6 months post operation. Representative three-dimensional reconstruction computed tomography of bone fusion volume in cases receiving the traditional method or novel device at 6 months post operation. The bone fusion areas around and within the cage were indicated in blue and yellow respectively and the cage pin was depicted in red

In the X-ray and computed tomography for efficacy assessment, non-union cases exhibited minimal bony incorporation and formation according to the X-ray and CT images obtained at 6 months post-surgery. Whereas the union cases showed bone bridge formation and the bone graft substitutes surrounding the cage, indicating solid spinal fusions (Fig. 4). We observed bony formation in the MIS device group as early as 3 months post operation compared with the control group, which did not exhibit comparable bony formation until later time points. The CT images 6 months after surgery also indicated greater bony incorporation in the MIS device group. Quantitatively, the MIS device group had significantly higher grade I and II fusion rates with no observable non-union cases throughout the follow-up period and at 24 months compared with the control group (Fig. 5). We found a grade I fusion rate of 3.6% in the control group and 39.5% in the MIS device group at 3 months post-surgery (Table 3). The fusion rate in the MIS device group increased to 63.2%, 88.2% and 96.1% at month 6, 12, and final follow-up point respectively, whereas the fusion rate in the control group was 12.5%, 37.5% and 75.0% respectively. The 3D reconstruction CT imaging demonstrated considerably higher fusion volume in the MIS device group with an average of 4.98 ± 0.86 cm^3^ compared with the 0.88 ± 0.20 cm^3^ on average in the conventional approach group, suggesting that the MIS device delivered a sufficient amount of bone graft which facilitated the spinal fusion (Fig. 6).

**Table 3:** Preoperative and 3, 6, 12, and 24 months grade I score percentage of the two bone graft filling approaches

Grade I (%)	Control group	MIS device group
Pre-op	0.0	0.0
Post-op 3 M	3.6	39.5
Post-op 6 M	12.5	63.2
Post-op 12 M	37.5	88.2
Post-op 24 M	75.0	96.1

## Discussion

In this study, we have demonstrated an improved bone healing capacity and fusion time with increased volumes of bone grafts in a MIS-TLIF procedure by using our novel MIS bone graft delivery device in the comparison with traditional tool. MIS-TLIF has shown less intraoperative blood loss, reduced postoperative narcotic use, fewer complications, and shorter hospital stay as well as recovery time compared to traditional open TLIF [4]. MIS-TLIF is sometimes preferred over other minimally invasive LIF given the disease and patients’ conditions. For instance, a better ODI, VAS pain, and complication rate were reported in treating degenerative lumbar disease when compared with MIS lateral lumbar interbody fusion (MIS-LLIF) [14]. Research also showed that MIS-TLIF is especially advantageous for obese patients [15]. One of the potential risks of MIS-TLIF is the inserted cannula that can damage the endplate or skate off to an undesired location. Repeat filling of the bone graft using traditional cannula also increases the risk of iatrogenic injury. Under the same minimally invasive procedure, the MIS device used in this study significantly reduced the filling time to only 20 s in addition to the subsequent blood loss volume. We also observed less blood loss when using the MIS device during the procedure.

Postoperative fusion rate along with clinical satisfaction indexes has been the gold standard of assessing LIF surgery efficacy. The postoperative fusion rate ranges from 70 to 95% depending on the patient-based factors, surgical techniques, and graft materials [16–20]. Although evidence have shown that graft materials influence LIF fusion rate, TLIF generally yields a high fusion rate despite the graft materials used [21]. A substantial effort was put for better postoperative lumbar stability which is reported to be related to the fusion rate [22]. The articulating cages or expandable cages give the surgeon better insertion control and increased footprint size on endplate and anterior contact in the disc space during MIS-TLIF. The use of these cages resulted in longer-lasting disc height restoration, improved lumbar lordosis and stability, providing a stable fusion environment [23, 24]. The relation between solid fusion and postoperative lumbar stability provided by pedicle screws were also investigated. While no surgical or clinical outcomes differences were found, Ren et al. demonstrated that bilateral pedicle screw fixation is preferred over unilateral fixation for less cage migration and higher fusion rate [25]. We used bilateral pedicle screw fixation for greater postoperative lumbar stability in this study, minimizing the possibility of skewed fusion rate due to lumbar instability.

To achieving successful bone fusion, the three pillars of bone regeneration including osteogenesis, osteo-induction, and osteo-conduction are critical [26]. The amount of transplanted bone graft serving the 3 properties is thus relatable to the fusion rate. Indeed, DiGiovanni et al. recently demonstrated that an adequate volume of bone graft between the osseous surface was needed for successful fusion in their ankle fusion study [7]. Although it is logical to assume the criticalness of adequate bone graft volume in lumbar fusion surgeries, the principle was only confirmed in MIS-TLIF by Yoo et al. [8]. Given the fact that less than 50% of the disc area is actually grafted in most LIF surgeries, increasing the graft volume transplanted is believed to be the desired strategy [27].

As the concept was demonstrated relatively recent, only a few studies have explored the possibility of transplanting greater bone graft volume using novel devices in LIF surgeries. An in-situ cage filling system was investigated using an in vitro lateral lumbar interbody fusion (LLIF) model, in which additional bone graft could be delivered after the pre-packed cage was inserted [28]. The delivery system significantly increased the cage volume with graft compared with the traditional cage filling methodology, however, it was difficult to predict the functionality of the novel system in a living human spine. The compressible liquid consistency of the bone graft poses a challenge during the filling process as the pressure applied by the plunger usually preferentially drives out the liquid part of the mixture. Kleiner et al. thus aimed to improve the graft flow and the volume delivered to the disc space by using a modified cannula during the MIS-TLIF [10]. The enlarged cross-sectional area of the cannula with outlets on the sides improved the flow of bone graft, allowing complete filling of the disc space. The authors, however, emphasized the relationship between the disk material removed and the graft volume for better graft volume prediction required during the procedure. The amount of bone graft in effect of fusion rate remained unknown. In this study, we integrated the threaded rod into the cannula that surgeons can connect to an electric surgical drill. The threaded rod concept not only resolved the shortfall of manual plungers that liquid graft mixture being the primary transplanted part, it also provided a stable and continuous filling process when in combination with the surgical drill. The MIS device delivered approximately 3 times more of the graft than that of using traditional funnel and cannula.

Notably, our study showed relatively small graft volumes were transplanted to the disc space compared with Yoo et al. [8] and Kleiner et al.’s studies [10]. Yoo et al. indicated a graft volume > 12 mL is considered to be sufficient for improved fusion rate, while the average bone graft delivered in Kleiner’s study was 9.2 mL. However, we demonstrated a higher than average fusion rate for MIS-TLIF with only an average of 6.72 mL bone graft volume. The difference may result from the mechanical design of the cannulas. The voids between bone graft granules can be diminished or collapsed during graft insertion, leading to a skewed final volume. Instead of compressing the graft granules manually through syringe and cannula, the threaded rod design moves the graft through the cannula and to the disc space without collapsing the granules and thus resulting in a small transplanted graft volume.

In MIS fusion surgeries, the biological environment may be different from the traditional open fusion surgeries, which are inherently more advantageous for fusion due to greater surface area and bigger cage for more graft materials. While the limitation of the MIS technique to completely remove disc space was reported to compromise the fusion rate, MIS-TLIF results in comparable outcomes as open TLIF in terms of solid fusion [29, 30]. Our study not only showed a higher fusion rate with the MIS device than other MIS-TLIF studies but also reaffirmed the notion of improved fusion rate with increased bone graft volume. Furthermore, this study is the first of its kind to elaborate the relationship between transplanted graft volume and the progression of fusion rate during the follow-up period. The use of the MIS device resulted in greater graft volume transplanted, leading to more than 88% fusion rate in the first year whereas the conventional methodology yielded only 75% solid fusion at the 2-year time point.

## Conclusions

The use of novel MIS bone graft delivery device significantly improves bone healing capacity and bone fusion time by increasing the amount of bone graft in patients receiving MIS-TLIF. The MIS device delivered 3 times more bone graft into the disc space and significantly reduced the intraoperative blood loss as well as the filling time to 20 s. This technique provides an efficient, safe, and time-saving surgical approach for surgeons in the field of MIS lumbar fusion surgery.

## Supplementary Information

**Additional file 1.** Patient data in this study.

## Data Availability

All the required information is contained in the manuscript. Further details can be requested by contacting the responding author Sher-Wei Lim (slsw0219@gmail.com).

## References

[1] Mobbs RJ, Phan K, Malham G, Seex K, Rao PJ (2015). Lumbar interbody fusion: techniques, indications and comparison of interbody fusion options including PLIF, TLIF, MI-TLIF, OLIF/ATP, LLIF and ALIF. J Spine Surg.

[2] Albert TJ, Pinto M, Denis F (2000). Management of symptomatic lumbar pseudarthrosis with anteroposterior fusion. A functional and radiographic outcome study. Spine (Phila Pa 1976).

[3] Salehi SA, Tawk R, Ganju A, LaMarca F, Liu JC, Ondra SL (2004). Transforaminal lumbar interbody fusion: surgical technique and results in 24 patients. Neurosurgery.

[4] Humphreys SC, Hodges SD, Patwardhan AG, Eck JC, Murphy RB, Covington LA (2001). Comparison of posterior and transforaminal approaches to lumbar interbody fusion. Spine (Phila Pa 1976).

[5] Hsieh PC, Koski TR, O'Shaughnessy BA, Sugrue P, Salehi S, Ondra S, Liu JC (2007). Anterior lumbar interbody fusion in comparison with transforaminal lumbar interbody fusion: implications for the restoration of foraminal height, local disc angle, lumbar lordosis, and sagittal balance. J Neurosurg Spine.

[6] Lowe TG, Tahernia AD, O'Brien MF, Smith DA (2002). Unilateral transforaminal posterior lumbar interbody fusion (TLIF): indications, technique, and 2-year results. J Spinal Disord Tech.

[7] DiGiovanni CW, Lin SS, Daniels TR, Glazebrook M, Evangelista P, Donahue R, Beasley W, Baumhauer JF (2016). The importance of sufficient graft material in achieving foot or ankle fusion. J Bone Joint Surg Am.

[8] Yoo JS, Min SH, Yoon SH (2015). Fusion rate according to mixture ratio and volumes of bone graft in minimally invasive transforaminal lumbar interbody fusion: minimum 2-year follow-up. Eur J Orthop Surg Traumatol.

[9] Chang KY, Hsu WK (2018). Spinal biologics in minimally invasive lumbar surgery. Minim Invasive Surg.

[10] Kleiner JB, Kleiner HM, Grimberg EJ, Throlson SJ (2016). Evaluation of a novel tool for bone graft delivery in minimally invasive transforaminal lumbar interbody fusion. Med Devices (Auckl).

[11] Shau DN, Parker SL, Mendenhall SK, Zuckerman SL, Godil SS, Devin CJ, McGirt MJ (2015). Transforaminal lumbar interbody graft placement using an articulating delivery arm facilitates increased segmental lordosis with superior anterior and midline graft placement. J Spinal Disord Tech.

[12] Clinicaltrials.gov, U.S. National Library of Medicine. Evaluation of Treatment Effect of Minimally Invasive Spinal Fusion Surgery. 2022. https://clinicaltrials.gov/ct2/show/study/NCT05190055.

[13] Ito Z, Imagama S, Kanemura T, Hachiya Y, Miura Y, Kamiya M, Yukawa Y, Sakai Y, Katayama Y, Wakao N, Matsuyama Y, Ishiguro N (2013). Bone union rate with autologous iliac bone versus local bone graft in posterior lumbar interbody fusion (PLIF): a multicenter study. Eur Spine J.

[14] Keorochana G, Setrkraising K, Woratanarat P, Arirachakaran A, Kongtharvonskul J (2018). Clinical outcomes after minimally invasive transforaminal lumbar interbody fusion and lateral lumbar interbody fusion for treatment of degenerative lumbar disease: a systematic review and meta-analysis. Neurosurg Rev.

[15] Tan JH, Liu G, Ng R, Kumar N, Wong HK, Liu G (2018). Is MIS-TLIF superior to open TLIF in obese patients?: A systematic review and meta-analysis. Eur Spine J.

[16] Schoenfeld AJ, Thomas D, Bader JO, Bono CM (2013). Transforaminal lumbar interbody fusion: prognostic factors related to retention in an active duty military population. Mil Med.

[17] von der Hoeh NH, Voelker A, Heyde CE (2017). Results of lumbar spondylodeses using different bone grafting materials after transforaminal lumbar interbody fusion (TLIF). Eur Spine J.

[18] Kim SS, Denis F, Lonstein JE, Winter RB (1990). Factors affecting fusion rate in adult spondylolisthesis. Spine (Phila Pa)..

[19] Formica M, Vallerga D, Zanirato A, Cavagnaro L, Basso M, Divano S, Mosconi L, Quarto E, Siri G, Felli L (2020). Fusion rate and influence of surgery-related factors in lumbar interbody arthrodesis for degenerative spine diseases: a meta-analysis and systematic review. Musculoskelet Surg.

[20] Snider RK, Krumwiede NK, Snider LJ, Jurist JM, Lew RA, Katz JN (1999). Factors affecting lumbar spinal fusion. J Spinal Disord.

[21] Galimberti F, Lubelski D, Healy AT, Wang T, Abdullah KG, Nowacki AS, Benzel EC, Mroz TE (2015). A systematic review of lumbar fusion rates with and without the use of rhBMP-2. Spine (Phila Pa 1976).

[22] Boden SD, Sumner DR (1995). Biologic factors affecting spinal fusion and bone regeneration. Spine (Phila Pa 1976).

[23] Hawasli AH, Khalifeh JM, Chatrath A, Yarbrough CK, Ray WZ (2017). Minimally invasive transforaminal lumbar interbody fusion with expandable versus static interbody devices: radiographic assessment of sagittal segmental and pelvic parameters. Neurosurg Focus.

[24] Cannestra AF, Peterson MD, Parker SR, Roush TF, Bundy JV, Turner AW (2016). MIS expandable interbody spacers: a literature review and biomechanical comparison of an expandable MIS TLIF with conventional TLIF and ALIF. Spine (Phila Pa 1976).

[25] Ren C, Qin R, Sun P, Wang P (2017). Effectiveness and safety of unilateral pedicle screw fixation in transforaminal lumbar interbody fusion (TLIF): a systematic review and meta-analysis. Arch Orthop Trauma Surg.

[26] Kalfas IH (2001). Principles of bone healing. Neurosurg Focus.

[27] Sukovich W (2004). Progress, challenges and opportunities in disc space preparation for lumbar interbody fusion. Internet J Spine Surg.

[28] Ozgur BM, Gillard DM, Wood EE, Truong FD, Wendel TG (2018). Can the use of a novel bone graft delivery system significantly increase the volume of bone graft material in a lumbar in situ cage, beyond volumes normally achieved via standard cage filling methodology? Results from a cadaveric pilot study. Interdiscip Neurosurg Adv Tech Case Manag.

[29] Qin R, Liu B, Zhou P, Yao Y, Hao J, Yang K, Xu TL, Zhang F, Chen X (2019). Minimally invasive versus traditional open transforaminal lumbar interbody fusion for the treatment of single-level spondylolisthesis grades 1 and 2: a systematic review and meta-analysis. World Neurosurg.

[30] Yao YC, Lin HH, Chou PH, Wang ST, Chang MC (2019). Differences in the interbody bone graft area and fusion rate between minimally invasive and traditional open transforaminal lumbar interbody fusion: a retrospective short-term image analysis. Eur Spine J.

